# Emergency care for young people after self-harm: a realist review protocol

**DOI:** 10.1136/bmjopen-2025-099554

**Published:** 2025-03-15

**Authors:** Daniel Romeu, Faye Ambler, Cathy Brennan, Judy M Wright, Andrew Booth, David Cottrell, Elspeth Guthrie

**Affiliations:** 1 University of Leeds, Leeds, UK; 2 The University of Sheffield, Sheffield, England, UK

**Keywords:** Suicide & self-harm, Child & adolescent psychiatry, ACCIDENT & EMERGENCY MEDICINE, Paediatric A&E and ambulatory care, Community child health, Review

## Abstract

**Introduction:**

In England, increasing numbers of young people seek help from emergency healthcare services, such as ambulances and emergency departments, after they self-harm. One contributing factor is a lack of meaningful and available community-based alternative sources of support for self-harm. It is not clear what helps young people in this context, how or why. This research aims to understand which resources are available in the emergency setting for young people (aged ≤25 years) who self-harm in England, and how and why they produce their intended and unintended effects.

**Methods and analysis:**

A realist review is a theory-driven interpretive approach to evidence synthesis. It provides realist logic of inquiry to produce an explanatory analysis of how and why resources work, for whom and in what circumstances. This review has two key components; one will identify the resources available in England for young people who self-harm in the emergency setting, the other will identify initial programme theories from the international literature. The review will closely follow Pawson’s five iterative stages: (1) clarifying scope, (2) evidence search, (3) article selection, (4) data extraction and organisation, and (5) evidence synthesis. Published and grey literature will be reviewed and included. Three key stakeholder groups will be involved throughout the review process, namely two patient and public involvement (PPI) groups (one for young people, one for parents and carers) and an interdisciplinary group of healthcare professionals.

**Ethics and dissemination:**

Ethical approval is not required for this review. Results will be reported according to Realist And Meta-narrative Evidence Synthesis: Evolving Standards publication and quality standards. Findings will be disseminated via a peer-reviewed publication in a scientific journal, conference presentations, a study website, an animated video shared via social media and other avenues identified by our PPI groups.

**PROSPERO registration number:**

CRD42025638539.

**STRENGTHS AND LIMITATIONS OF THIS STUDY**

- A comprehensive search strategy has been developed with a senior information specialist to capture the most relevant literature; this includes systematic searches of electronic databases and grey literature sources, and strategies such as citation searching and snowballing.
- Our review includes contributions from three key stakeholder groups, namely two separate patient and public involvement groups (one for young people, one for caregivers of young people who self-harm) and an interdisciplinary advisory group of diverse healthcare professionals who work with young people in different settings.
- The inclusion of multiple stakeholder groups may create issues in reaching consensus and in configuring, consolidating and prioritising programme theories.
- Our review is exploratory and iterative in nature; it may be limited by publication bias and the richness and relevance of evidence available in the literature.
- Only articles written in the English language will be included, representing a limitation and source of language bias.

## Background

Self-harm refers to any intentional self-injury or self-poisoning, regardless of intent,^1^ and it encompasses a broad spectrum of behaviours with diverse functions.^2^ It is common in young people, with one-quarter of 17-year-olds in the UK having self-harmed at least once in the previous 12 months^3^. Self-harm is a significant public health concern; it is the single best predictor of suicide,^4^ a key priority of the National Health Service (NHS) Long-Term Plan^5^ and ‘everybody’s business’ according to the National Institute for Health and Care Excellence (NICE) guidance^1^ (table 1).

**Table 1:** Definition of terms

Resource	Given this review’s exploratory aim and its focus on the complicated and diverse landscape of mental health programmes, interventions and services, the term ‘**resource**’ will be used to capture **anything (economic, material, emotional, social)** that might be offered to a young person after they self-harm**.**
Context	Greenhalgh and Manzano identify two distinct but overlapping conceptualisations of “context” in realist research,^25^ both referring to background features that interact with mechanisms to shape how and why an intervention works (or not): Tangible, observable and static features or things (eg, demographics, policy, geographical setting) that shape a mechanism. Relational, emergent and dynamic features or forces (eg, interpersonal relationships, institutional settings, cultural norms) that shape a mechanism.
Mechanism	The underpinning generative force that leads to outcomes (both intended and unintended), usually divided into two constituent parts^27^: The **resources** offered by an intervention (formal and informal). How people **respond to** and **reason with** those resources.
Outcome	The measurable impact (intended or unintended) at the behavioural, clinical or system level, based on context-mechanism interactions.
Context-mechanism-outcome configuration	An analytical tool within the realist approach which aims to articulate what works, for whom, how and why, and in what circumstances.For example: *Young people present to hospital-based mental health crisis teams following a self-harm episode (**context**). If crisis team policy requires clinicians to follow-up every patient every 2 days (**mechanism resource**), then patients will gain the sense that they are not alone and that somebody cares about their well-being (**mechanism response**), leading to a reduction in self-harm ideation (**outcome**)*.
Initial programme theory	A hypothetical statement, often in the form of “if … then”, that is developed at the start of a realist synthesis or evaluation, to explain how a programme or programme component is thought to work (or not work).For example: ***If** a young person calls a crisis telephone line when they are experiencing self-harm ideation, and the call handler calmly encourages them to engage in mindfulness and breathing exercises, **then** they will feel supported, increasing the likelihood of the young person engaging in such exercises, leading to somatic relaxation and improved emotional processing*.
Rival theory	A hypothetical statement that shows how the same programme resources can lead to different (even opposite) responses and outcomes.For example: ***If** a young person calls a crisis telephone line when they are experiencing self-harm ideation, and the call handler calmly encourages them to engage in mindfulness and breathing exercises, **then** they will feel that the call handler is minimising the intensity and complexity of their feelings and not adapting their approach to the young person’s specific needs, leading to a sense of not feeling listened to and subsequent frustration, increasing the likelihood of engaging in self-harm*.
Retroduction	A form of reasoning that moves between empirical observations and theoretical explanations to identify the underlying causal mechanisms and structures that generate observed patterns or regularities. It combines elements of both inductive and deductive reasoning but goes beyond them by seeking to explain what must be true for observed phenomena to occur.
RAMESES	Acronym for ‘Realist And Meta-narrative Evidence Synthesis: Evolving Standards’, two National Institute for Health and Care Research-funded projects aiming to produce quality and publication standards and training materials for realist research approaches.^45^

Internationally, options for young people seeking emergency care following self-harm include emergency departments (EDs), specialist community mental health teams, school services, social care initiatives, charities and helplines.^6^ In England, there is a growing focus on collaborative working between healthcare and other services, but this has not materialised in practice. Waiting lists for specialist child and adolescent mental health services (CAMHS) vary significantly across the country and sometimes exceed 2 years.^7^ Some regions only provide specialist services within office hours.^8^

Increasing numbers of young people are attending hospital EDs after self-harm.^9^ They report feeling let down by the healthcare system, only attending the ED because appropriate alternatives are lacking.^10^ Assessment in hospital is not always necessary, and often the busy environment can have negative implications on the young person’s mental state.^11^ There are often long waits to be seen, and frontline staff such as ambulance^12^ and ED^11^ clinicians lack training and confidence in managing mental health presentations.

There is a paucity of evidence linking emergency interventions for young people who self-harm with outcomes. A recent Cochrane review of psychosocial interventions for young people who self-harm only identified low-quality evidence from 17 trials.^13^ Nonetheless, there are national standards of care for young people experiencing acute mental health difficulties^14^; for example, care should be immediately available and community-based wherever possible. Recent national implementation guidance from NHS England also emphasises multiagency working and hospital prevention as important guiding principles.^15^

Despite the existence of national standards, it is still not clear what young people find helpful when seeking support immediately after they self-harm: a better understanding of this is important to inform evidence-based decision-making and therefore influence policy and commissioning. By summarising which resources exist and how people respond to them, it may be possible to adapt existing services or develop new ones to improve outcomes for young people at regional and national levels. A realist approach is an appropriate methodological choice when exploring such information.^16^

Realist reviews use theory to explore how contexts, such as societal norms and service infrastructure, interact with underlying mechanisms to produce outcomes, both intended and unintended.^17^ They reveal important information about the effectiveness and mechanism of different resources, enabling service providers and clinicians to design and implement services or interventions comprising only effective components for particular people in particular contexts.^18^ Medical Research Council guidance suggests that programme theories facilitate the inter-setting transferability of interventions and the production of evidence that is useful to decision-makers.^19^

## Objectives

The aim of this research is to understand **which** resources (anything that might be offered by a programme, intervention, service or individual) are available in the emergency setting for young people (aged ≤25 years) who self-harm in England, and **how** and **why** they produce their effects, both intended and unintended.

The research questions are as follows:

1. What efforts exist in the emergency setting across England to provide young people with a positive and helpful experience after they self-harm? (**mechanism resources+outcomes**)
2. How do these efforts and initiatives help young people? (**mechanism responses+outcomes**)
3. What are the barriers and enablers to providing emergency care for young people after they self-harm? (**context**)

## Methods and analysis

Realist review using systematic methods comprising two components (mapping component and theory-building component), with distinct but overlapping search strategies. The protocol is registered on PROSPERO: CRD42025638539. The completed Preferred Reporting Items for Systematic Review and Meta-Analysis Protocols checklist^20^ can be found in online supplemental file 1.

Pilot searches have confirmed the originality and feasibility of this review. In the context of long waiting lists^7^ for specialist children’s mental health services, this review will be helpful in synthesising the evidence base to identify the principles of providing effective and timely care in the emergency setting for young people after they self-harm.

### Study status

Study start date: August 2024.

Expected end date: January 2026.

At the time of writing, the study status is as follows:

- Clarifying scope: started.
- Search strategies: started.
- Title and abstract screening: started.
- Full-text screening: not started.
- Data extraction: not started.
- Quality assessment: not started.
- Data analysis and synthesis: not started.

### Realist review

A realist review is an interpretive, theory-driven approach^21^ to evidence synthesis from multiple sources, such as published research, policy documents and grey literature.^17^ The realist approach acknowledges that resources work in some contexts and not others, and for some people but not others. It applies realist logic of inquiry to produce an explanatory analysis of what a resource is, how it works, for whom and in what circumstances.^22^ Realist reviews are typically used to understand complex interventions,^21^ comprising multiple components and outcomes and long pathways to the desired outcome(s).^23^

Realist reviews are retroductive, focusing on identifying underlying causal mechanisms, with causation being represented as context+mechanism=outcome.^24^ **Context** refers to ‘background’ features that interact with mechanisms to shape how and why interventions work (or not); they can be tangible and static features (eg, demographics, policy, geographical setting) or relational and dynamic features (eg, interpersonal relationships, cultural norms).^25^ The realist approach recognises micro (individual), meso (organisational) and macro (systemic) contexts.^26^ **Mechanisms** refer to causal forces that are activated in particular contexts to bring about outcomes. They explain how and why observed outcomes occur and usually comprise two parts, the ‘resources’ offered by an intervention, and the cognitive, emotional and/or behavioural ‘reaction’ or ‘response’ to the resource.^27^ **Outcomes** are the intended or unintended effects of the intervention, which are generated by the interaction between context and mechanism.^23^

One of the central processes in a realist review is the development of **programme theories**, referring to hypotheses for what a programme comprises and how it is expected to work.^23^ Programme theories are particularly useful for complex and varied programmes, interventions and services which are context-sensitive,^19^ such as is the case in mental healthcare. They are conventionally presented as context-mechanism-outcome configurations (CMOCs), an analytical tool intended to gain generative causal understanding of the most important resources on offer.^28^

Stakeholder engagement throughout the realist review process is encouraged to promote the inclusion of multiple perspectives.^24^ Three stakeholder groups will be actively engaged in the review process: one interdisciplinary group of healthcare professionals working clinically with young people who self-harm, and two patient and public involvement (PPI) groups, one for young people and one for parents and carers of young people who self-harm. Stakeholder groups will help identify and refine initial programme theories through discussions via emails and workshops (remote or in-person, according to individual preferences).

At present, there is little understanding of how and why different resources lead to particular outcomes for young people who self-harm. The realist review will not provide a summative judgement on whether particular resources are ‘good’ or ‘bad’, but will instead explain how and why they work, in what contexts, for whom and to what extent.

Reporting standards for realist syntheses exist, although specific methods for conducting them vary.^29^ Pawson and colleagues outline five stages of realist synthesis^17^ which will be followed in this review. The review design and methods are explained in detail below.

#### Clarifying scope

As a first step, we will carry out exploratory, informal searches of the published and grey literature to identify initial programme theories and a draft programme architecture. The exploratory searching of Step 1 differs from the formal data searches outlined in Step 2, in that it aims to sample the literature to quickly identify the diversity of possible theories and resources. Relevance will be prioritised over methodological rigour.

These searches will be supplemented by consulting with key stakeholder groups and topic experts. This will be achieved through a combination of stakeholder meetings and email exchanges. Formal ethical approval will not be required but informed participation will be sought.

For this review, the term ‘resource’ will be used to refer to anything (economical, material, emotional, social) that might be offered to a young person in England immediately after they self-harm. Sources of these resources are likely to include

- NHS telephone lines (111, mental health crisis lines)
- NHS walk-in centres and urgent care centres
- Ambulances
- EDs
- Specialist mental health services (CAMHS, adult crisis services)
- Non-NHS text-based services
- Non-NHS telephone lines (eg, Samaritans)
- Education-based support (school, university)
- Non-NHS community-based support (charities, crisis cafes, safe spaces)
- Emergency social care interventions

Building a set of initial programme theories will require iterative discussions within the team and with key stakeholder groups and topic experts.

#### Search strategies

Two distinct but overlapping search strategies will be conducted and continually refined, in line with the realist approach:^30^

- **Strategy 1** will identify the initial programme theories from the international literature (both published and grey). Suitable literature will include qualitative research, service reports, think pieces and theory-driven literature.
- **Strategy 2** will identify the resources available in the emergency setting to young people who self-harm in England. It will identify routinely offered services and interventions, as well as examples of current best practice, pilots and other relevant initiatives. There will be a focus on the interface between NHS services and community-based psychosocial interventions.

We will search the following electronic databases from 2004 (coinciding with the publication of the first NICE Guideline, CG16, on the management of self-harm in over 8s^31^) to 2 December 2024: MEDLINE, PsycINFO, Embase, HMIC, CINAHL, Science and Social Sciences Citation Index and The Cochrane Library. Search strategies were co-developed with a senior information specialist (JMW) and translated across databases using Polyglot.^32^ See onlinesupplemental files 2  3 for theory-building and mapping search strategies for MEDLINE.

Targeted grey literature searches (ProQuest Dissertations and Theses, Google Scholar) will identify other relevant literature, such as opinion pieces, books, guidelines, policies, editorials and dissertations. In addition, the following methods will be used to identify relevant evidence from diverse sources for inclusion in the review:

- A Google Scholar search will be conducted to ensure that key results are not missed. After ranking by relevance, the top 100 results will be screened. This will be facilitated by Publish Or Perish.^33^
- Reference lists from relevant primary studies and systematic reviews will be checked (snowballing).
- Citation searches, for example, using the ‘Cited by’ option on Google Scholar, and/or Publish Or Perish^33^ (lateral searching).
- Input will be sought from the review team and stakeholder advisory groups to uncover other relevant publications, guidelines and policies.

Specific website searches will also be conducted; these have been selected based on input from key stakeholder groups, topic experts and relevant service providers:

- https://www.mentalhealth.org.uk/
- https://www.rcpch.ac.uk/
- https://www.rcpsych.ac.uk/
- https://rcem.ac.uk/
- https://www.rcgp.org.uk/
- https://www.rcn.org.uk/
- https://collegeofparamedics.co.uk/
- https://www.nhs.uk/
- https://www.youngminds.org.uk/
- https://www.samaritans.org/
- https://www.mind.org.uk/
- https://nspa.org.uk/
- https://www.barnardos.org.uk/
- https://www.papyrus-uk.org/
- https://www.selfharm.co.uk/
- https://www.selfinjurysupport.org.uk/
- https://sossilenceofsuicide.org/
- https://www.nspcc.org.uk/
- https://www.place2be.org.uk/
- https://www.mindwell-leeds.org.uk/
- https://sites.manchester.ac.uk/mash-project/support-for-improving-community-based-care-for-self-harm/
- https://www.gov.uk/
- https://committees.parliament.uk/publications/
- https://www.yas.nhs.uk/
- https://www.samaritans.org/
- https://www.bacp.co.uk/

The realist approach to evidence searching is iterative,^30^ focusing on identifying relevant programme theories and testing them against empirical data. It is acknowledged that realist search strategies aim to uncover fragmented data; search strategies will therefore be iteratively extended and refocused as the review progresses. This may involve purposive sampling and snowballing to confirm, refine or refute the theories as new evidence emerges.

All retrieved records will be imported into EndNote^34^ for organisation and de-duplication, before transferring to Rayyan^35^ to facilitate title and abstract screening.

Titles and abstracts, where available, will be screened to assess eligibility for full-text inclusion. Eligibility criteria for the main search will be broad to ensure identification of qualitative, quantitative and mixed methods studies. Table 2 summarises the inclusion and exclusion criteria we have developed to focus on the review, although these are likely to be refined and updated as the review progresses, and as programme theories are developed. Given the anticipated high volume of relevant literature, additional criteria may be added in line with stakeholder group feedback.

**Table 2:** Review inclusion and exclusion criteria

	Strategy 1 (theory-building)	Strategy 2 (mapping)
Inclusion criteria		
Population (P)	Young people (aged ≤25 years) who self-harm and/or any of their caregivers (eg, family, friends, partners, etc)Any professional who provides support to young people after they self-harm (eg, doctors, nurses, paramedics, social workers, support workers, volunteers, etc)	Young people (aged ≤25 years) who self-harm
Intervention (I)	Any programme, service, intervention or initiative, including routinely offered services, examples of best practice and pilots	Any programme, service, intervention or initiative, including routinely offered services, examples of best practice and pilots
Comparator (C)	None	None
Outcome (O)	Outcomes of interest will depend on the intervention but could include any measurable impact (intended or unintended) on young people, their caregivers, healthcare professionals and/or healthcare services	None
Healthcare context (H)	Any urgent or emergency setting, or anything between act of self-harm and access to support	Any urgent or emergency setting, or anything between act of self-harm and access to support
Design	No restriction	No restriction
Location	Worldwide (but only in English)	England only
Exclusion criteria		
	Non-English papersStudies in non-emergency settings, such as within-hours primary care, inpatient wards and prison settings	Self-management strategies (eg, mobile phone apps)

All citations will be reviewed by DR to determine if they match the eligibility criteria. For Strategy 1, a random sample of 10% of all citations will be reviewed independently by FA to ensure consistency around the application of the eligibility criteria. However, in cases of uncertainty, discussion with a third reviewer (CB) will be used to prevent premature exclusion of potentially pivotal papers. For Strategy 2, all citations will be independently screened by FA, given the objectivity of anticipated findings. Disagreements will be resolved through discussion with a third reviewer (CB) to ensure consistency in paper inclusion.

#### Selecting articles

In line with the realist approach, quality assessments of the full-text articles will be completed according to three criteria: relevance, richness and rigour.^36^ Documents will be selected for coding based on their relevance to contributing to an understanding of which resources are available in the emergency setting for young people who self-harm in England, and how and why they produce their effects (both intended and unintended).

Having completed the eligibility screening, DR will screen the full texts of all articles retrieved by the formal searches for relevance and richness. Criteria from the published literature^18^ will be adapted and used to rank the relevance and conceptual richness of studies to help with the study selection process. A random sample of 10% of documents selected will be independently assessed for relevance by FA to ensure that screening and selection decisions are made consistently. Any disagreements will be resolved through discussion with a third reviewer (CB).

Table 3 summarises the ranking criteria for relevance that will allow the review team to distinguish between conceptually rich and weaker evidence to achieve the review’s aims. This is likely to be developed iteratively throughout the review process.

**Table 3:** Criteria to rank likely relevance of study to theory identification and development

High relevance	Relates to young people who self-harm and describes the implementation of programmes, services, interventions and/or initiatives, or describes the provision of resources in the emergency setting Describes the perspectives and factors affecting the decision-making of young people seeking emergency care for self-harm and/or their caregivers Relates to supporting young people who self-harm and includes descriptions of professional views and experiences of providing support Relates to managers and/or commissioners of programmes, services, interventions and/or initiatives involving the provision of resources to young people who self-harm Describes training of practitioners who provide care to young people who have self-harmed in the emergency setting
Moderate relevance	Relates to young people who self-harm and describes their experiences of interacting with resources provided in the emergency setting Describes experiences of young people who self-harm and/or caregivers who have chosen not to seek help or support immediately after an act of self-harm Describes young people’s support needs after self-harm
Low relevance	Quantitative data on programmes, services, interventions and/or initiatives for young people who self-harm in the emergency settings Describes implementation and/or delivery of programmes, services, interventions and/or initiatives for young people who self-harm at other stages of their journey (ie, not the emergency setting)
No relevance	Does not meet any of the above criteria

Rigour will be assessed with reference to credibility and trustworthiness, as outlined by Realist And Meta-narrative Evidence Synthesis: Evolving Standards (RAMESES).^23^ Central to the realist approach is that a conventional ‘hierarchy of evidence’ is not applicable as valuable causal insights for programme theory development can arise from seemingly poor quality studies.^37^ We will therefore consider evidence of lesser quality if relevant for identifying and developing programme theories and/or resources on offer to young people in England after they have self-harmed. A realist synthesis appraisal form will be developed on Google Forms by adapting an existing template,^38^ and this will be completed for each article. Specific design limitations will be documented where identified and caveats will be included in the narrative results.

Depending on the number of papers included, further refinement of the review scope may be decided by the review team. Any decisions regarding additional searches will depend on whether they are anticipated to contribute to the review’s aims.

#### Extracting and organising data

Once article selection has been finalised and the core data set established, DR will re-read the full texts of the included articles in reverse chronological order and carry out initial categorical coding. During this familiarisation stage, an analytical journal will be completed in parallel, outlining potential contexts, mechanisms, outcomes and configurations, as well as reflections on the ‘big picture’ that emerge through the data set. Bespoke Excel data extraction forms will be developed for both searches, based on examples in the literature.^39^

The theory-building data extraction tool (theory-building component) will include sections for study design, sample, resources and potential contexts (C), mechanisms (M) and outcomes (O) to aid interpretation and facilitate the identification of programme theories. As per the realist approach, data will focus on author explanations and discussions about how a particular resource was thought to work (or not). Individual papers may include segments that contribute to different parts of a programme theory. DR will then re-read the data set, extract relevant data segments and collate them into the corresponding sections of the theory-building data extraction tool. A random sample of 10% of documents selected will be independently reviewed and data extraction by FA to ensure consistency. DR will continue to complete the analytical journal throughout to enable contemporaneous documentation of how data has contributed to theory-building.

The mapping component data extraction tool will summarise key study information including study aims, design and methods, study participants, setting and staff. Given the objectivity of the anticipated findings, all citations will be independently screened by FA. Particular attention will be paid to gaps in resource provision and the consistency of funding and resource provision across the country. Resources identified will be broadly divided into healthcare, school-based, university-based, social care and third-sector organisations, although this will be determined and refined through exploration of the data.

#### Data synthesis

Electronic versions of all articles will be uploaded to NVivo 15^40^ for further analysis. The data within the data extraction forms will be re-read, and where appropriate, recoded and reclassified. Coding will be continually refined in NVivo and relationships (a NVivo function) will be used to create links between contexts, mechanisms and outcomes where possible across the data set.^41 42^ A combination of an inductive (codes emerging from the literature) and deductive (codes created in advance informed by programme theories, stakeholder discussions and exploratory literature searching) approach will be used. The reflective journal will continue to be completed in parallel. A retroductive logic of analysis will be used to analyse and synthesise the data throughout.

Having identified potential contexts, mechanisms, outcomes and CMOCs, analysis will continue iteratively using the realist inquiry of explanatory logic. Starting from relevant outcomes, we will seek to interpret and explain how different stakeholders respond to resources offered to a young person following self-harm and to identify the specific contexts or circumstances when relevant mechanisms are likely to be triggered. This analysis will be repeated throughout the review to enable the construction of CMOCs to explain how and why different resources offered in the emergency setting help young people after they self-harm (or not), and in what circumstances.

Data synthesis will involve reflection and discussion among the review team. We will question the integrity of each programme theory by examining whether it is supported by empirical evidence, adjudicate between competing theories, consider the same programme theory in different contexts and compare the programme theories to practical experiences of service users and providers.^43^

Identified initial programme theories will be presented to the three stakeholder advisory groups. These key informants will facilitate programme theory prioritisation for refinement and testing in future WPs, based on an a priori criterion of 70% stakeholder agreement.^44^ Advisory group discussions, outcomes and justifications will be captured as field notes.

The final output of this review will be a detailed summary of the nature and diversity of resources available in the emergency setting to young people in England after they self-harm, and a final realist programme theory, outlining how and why these resources produce their effects. Findings will be summarised through narrative synthesis, using text, summary tables, a logic model and, where appropriate, graphics to summarise individual papers and draw insights across papers. We acknowledge that this may represent partial knowledge due to the necessary prioritisation of programme theories and information sources limiting the ground that can be covered by a single review.^17^

### Patient and public involvement

Two PPI groups have been assembled to support this review and associated studies; one for young people with experience of self-harm and one for caregivers. PPI representatives were identified by contacting local charities, sharing information through relevant mailing lists and through existing PPI networks.

Members of the public were involved in the development of this protocol. Both PPI groups have reviewed this protocol and contributed to the grey literature search strategy. They will help identify and refine initial programme theories through discussions via emails and/or workshops (remote or in-person, according to their preferences).

## Ethics and dissemination

This review does not require ethical approval as no primary data will be collected or analysed.

Results will be reported according to the RAMESES quality and publication standards.^45^ Findings will be presented in a way that offers contextual advice rather than general conclusions. This allows policymakers to adapt resources to specific contexts, providing practical insights instead of ‘one-size-fits-all’ recommendations.

We will disseminate findings via a peer-reviewed article in a suitable academic journal, conference presentations, a report to the funder (National Institute for Health and Care Research (NIHR)), a study website (in development), animated videos via social media and any other avenues identified by our PPI groups. Existing contacts with Integrated Care Boards, NHS England and clinical networks represent avenues for broader dissemination.

This review is being undertaken as part of the wider Emergency Care After Self-Harm (EmCASH) study, a mixed-methods realist synthesis and evaluation of emergency care for young people who self-harm in England. Findings will be used to inform the next stages of the project and have the potential to benefit multiple stakeholders involved in developing, implementing and evaluating sources of emergency care for young people who self-harm.

## supplementary material

10.1136/bmjopen-2025-099554 online supplemental file 1

10.1136/bmjopen-2025-099554 online supplemental file 2

10.1136/bmjopen-2025-099554 online supplemental file 3
