# Supplementary material for: Emergency care for young people after self-harm: a realist review protocol
Source: BMJ Open. 2025 Mar 15;15(3):e099554. doi: 10.1136/bmjopen-2025-099554 (PMC11911665; doi:10.1136/bmjopen-2025-099554)
Supplement: online supplemental file 2 [file bmjopen-15-3-s002.docx]

**EMERGENCY CARE FOR YOUNG PEOPLE AFTER SELF-HARM: A REALIST REVIEW PROTOCOL**

**Supplementary file 2.** Theory-building search strategy for MEDLINE.

**Ovid MEDLINE(R) ALL <1946 to January 14, 2025>**

1 Self-Injurious Behavior/ 10817

2 suicide/ or suicide, attempted/ or Suicide, Completed/ 61900

3 Drug Overdose/ 15535

4 Self Mutilation/ 3257

5 (selfharm* or selfinjur* or selfinflict*).tw,kf. 98

6 ((self or themsel* or onesel*) adj2 (aggress* or harm* or cutt* or immolat* or inflict* or injur* or mutilat* or poison* or damag* or destruct*)).tw,kf. 29883

7 (automutilat* or "auto mutilat*" or auto-mutilat*).tw,kf. 147

8 (autoaggress* or "auto aggress*" or auto-aggress).tw,kf. 1079

9 suicidality.tw,kf. 10422

10 (suicid* adj2 (death or die* or morality or complete)).tw,kf. 5138

11 (suicid* adj2 (attempt* or behavio* or intent* or intend* or commit*)).tw,kf. 36938

12 (parasuicid* or para-suicid*).tw,kf. 687

13 (poison adj2 (deliberat* or intentional or intended)).tw,kf. 19

14 (overdos* adj2 (deliberat* or intentional or intended)).tw,kf. 712

15 NSSI.tw,kf. 2303

16 or/1-15 [self harm] 119551

17 exp Community Health Services/ 341812

18 Crisis Intervention/ 6412

19 emergency medical services/ or call centers/ or emergency medical dispatch/ or emergency medical service communication systems/ or exp emergency service, hospital/ or emergency services, psychiatric/ or hotlines/ or poison control centers/ or exp "transportation of patients"/ 171576

20 exp emergency responders/ or paramedics/ 16628

21 ((phone* or call* or telephone* or "hot line*") adj5 service*).tw,kf. 6679

22 ("nhs 111" or helpline* or help-line*).tw,kf. 1501

23 (pre-hospital or prehospital).tw,kf. 23743

24 (ambulance* or paramedic*).tw,kf. 23538

25 (crisis adj5 (intervention* or service* or centre* or center* or cafe*)).tw,kf. 4731

26 (emergency adj5 (intervention* or service* or centre* or center* or department*)).tw,kf. 175707

27 "accident and emergency".tw,kf. 5293

28 (Emergency adj5 (technician? or assistant?)).tw,kf. 1963

29 or/17-28 [Emergency pre hospital setting] 640998

30 samaritans.tw. 150

31 touchstone.tw. 263

32 "battle scars".tw. 9

33 sane.tw. 1123

34 selfharmUK.tw. 0

35 "rethink mental illness".tw. 1

36 papyrus.tw. 423

37 calm.tw. 5010

38 "recover your life".tw. 0

39 "mental health matters".tw. 58

40 "self injury support network".tw. 0

41 or/30-40 [Self harm organisations] 7035

42 29 or 41 [NHS and other mental health service providers] 647618

43 triage/ 15908

44 Critical Pathways/ 8221

45 exp Decision Making/ 245050

46 pathway*.tw,kf. 1610187

47 (help adj1 seek*).tw,kf. 14238

48 exp "Delivery of Health Care"/ 1350526

49 "Health Services Needs and Demand"/ 55961

50 (demand* adj2 manage*).tw,kf. 1605

51 ((service or delivery) adj2 model*).tw,kf. 10748

52 (service? adj3 ("use" or used or utili#ation or utili#ed or utili#ing or access* or engage*)).tw,kf. 79648

53 health-care service*.tw,kf. 20972

54 health* service*.tw,kf. 187726

55 attend*.tw,kf. 241890

56 (present* adj3 (selfharm* or self-harm* or suicid*)).tw,kf. 2358

57 or/43-56 [Choosing or Accessing Services] 3488579

58 (policy or policies or guideline* or recommendation* or position).ti. 276177

59 guideline/ or practice guideline/ 39978

60 policy/ or public policy/ or exp health policy/ 178490

61 (theor* or concep* or logic).ti. 257021

62 ((theor* or concep* or logic) adj (framework* or model* or analy* or evaluat*)).ab. 109276

63 or/58-62 [Policy, Guideline or overt Theory] 773803

64 Comment/ 1046598

65 Letter/ 1284762

66 Editorial/ 717997

67 news/ or newspaper article/ 245899

68 "Comment on".ti. 38864

69 (letter* adj3 editor*).ti. 30813

70 opinion*.ti. 19573

71 (view or views).ti. 66312

72 or/64-71 [Discussion papers Hidden Theory] 2626202

73 63 or 72 [Theory Search] 3324702

74 (Literature review* or (systematic adj2 review*) or (narrative adj2 review*) or (critical adj2 review*) or scoping review* or synthesis or meta-analys* or ((realist adj2 review*) or meta-ethnograph*)).ti. 878833

75 ("review of reviews" or ((overview* or umbrella) adj5 review*)).ti. 4380

76 ("Search filter*" or "search strateg*" or "literature search*").ab. 112786

77 meta-analysis/ or "systematic review"/ 372619

78 or/74-77 [Systematic review search] 1000677

79 73 or 78 [Theory or Systematic review search] 4280597

80 16 and 42 and 57 and 79 [Theories or systematic reviews around access, choice or demand for health services and providers for self-harm] 364

81 limit 80 to yr="2004 -Current" 309
