# Supplementary material for: Emergency care for young people after self-harm: a realist review protocol
Source: BMJ Open. 2025 Mar 15;15(3):e099554. doi: 10.1136/bmjopen-2025-099554 (PMC11911665; doi:10.1136/bmjopen-2025-099554)
Supplement: online supplemental file 3 [file bmjopen-15-3-s003.docx]

**EMERGENCY CARE FOR YOUNG PEOPLE AFTER SELF-HARM: A REALIST REVIEW PROTOCOL**

**Supplementary file 3.** Mapping search strategy for MEDLINE.

**Ovid MEDLINE(R) ALL <1946 to January 14, 2025>**

1 Self-Injurious Behavior/ 10817

2 suicide/ or suicide, attempted/ or Suicide, Completed/ 61900

3 Drug Overdose/ 15535

4 Self Mutilation/ 3257

5 (selfharm* or selfinjur* or selfinflict*).tw,kf. 98

6 ((self or themsel* or onesel*) adj2 (aggress* or harm* or cutt* or immolat* or inflict* or injur* or mutilat* or poison* or damag* or destruct*)).tw,kf. 29883

7 (automutilat* or "auto mutilat*" or auto-mutilat*).tw,kf. 147

8 (autoaggress* or "auto aggress*" or auto-aggress).tw,kf. 1079

9 suicidality.tw,kf. 10422

10 (suicid* adj2 (death or die* or morality or complete)).tw,kf. 5138

11 (suicid* adj2 (attempt* or behavio* or intent* or intend* or commit*)).tw,kf. 36938

12 (parasuicid* or para-suicid*).tw,kf. 687

13 (poison adj2 (deliberat* or intentional or intended)).tw,kf. 19

14 (overdos* adj2 (deliberat* or intentional or intended)).tw,kf. 712

15 NSSI.tw,kf. 2303

16 or/1-15 [self harm] 119551

17 exp Community Health Services/ 341812

18 Crisis Intervention/ 6412

19 emergency medical services/ or call centers/ or emergency medical dispatch/ or emergency medical service communication systems/ or exp emergency service, hospital/ or emergency services, psychiatric/ or hotlines/ or poison control centers/ or exp "transportation of patients"/ 171576

20 exp emergency responders/ or paramedics/ 16628

21 ((phone* or call* or telephone* or "hot line*") adj5 service*).tw,kf. 6679

22 ("nhs 111" or helpline* or help-line*).tw,kf. 1501

23 (pre-hospital or prehospital).tw,kf. 23743

24 (ambulance* or paramedic*).tw,kf. 23538

25 (crisis adj5 (intervention* or service* or centre* or center* or cafe*)).tw,kf. 4731

26 (emergency adj5 (intervention* or service* or centre* or center* or department*)).tw,kf. 175707

27 "accident and emergency".tw,kf. 5293

28 (Emergency adj5 (technician? or assistant?)).tw,kf. 1963

29 or/17-28 [Emergency pre hospital setting] 640998

30 samaritans.tw. 150

31 touchstone.tw. 263

32 "battle scars".tw. 9

33 sane.tw. 1123

34 selfharmUK.tw. 0

35 "rethink mental illness".tw. 1

36 papyrus.tw. 423

37 calm.tw. 5010

38 "recover your life".tw. 0

39 "mental health matters".tw. 58

40 "self injury support network".tw. 0

41 or/30-40 [Self harm organisations] 7035

42 29 or 41 [NHS and other mental health service providers] 647618

43 triage/ 15908

44 Critical Pathways/ 8221

45 exp Decision Making/ 245050

46 pathway*.tw,kf. 1610187

47 (help adj1 seek*).tw,kf. 14238

48 exp "Delivery of Health Care"/ 1350526

49 "Health Services Needs and Demand"/ 55961

50 (demand* adj2 manage*).tw,kf. 1605

51 ((service or delivery) adj2 model*).tw,kf. 10748

52 (service? adj3 ("use" or used or utili#ation or utili#ed or utili#ing or access* or engage*)).tw,kf. 79648

53 health-care service*.tw,kf. 20972

54 health* service*.tw,kf. 187726

55 attend*.tw,kf. 241890

56 (present* adj3 (selfharm* or self-harm* or suicid*)).tw,kf. 2358

57 or/43-56 [Choosing or Accessing Services] 3488579

58 exp United Kingdom/ 401844

59 (national health service* or nhs*).ti,ab,in. 306886

60 (english not ((published or publication* or translat* or written or language* or speak* or literature or citation*) adj5 english)).ti,ab. 138546

61 (gb or "g.b." or britain* or (british* not "british columbia") or uk or "u.k." or united kingdom* or (england* not "new england") or northern ireland* or northern irish* or scotland* or scottish* or ((wales or "south wales") not "new south wales") or welsh*).ti,ab,jw,in. 2632240

62 (bath or "bath's" or ((birmingham not alabama*) or ("birmingham's" not alabama*) or bradford or "bradford's" or brighton or "brighton's" or bristol or "bristol's" or carlisle* or "carlisle's" or (cambridge not (massachusetts* or boston* or harvard*)) or ("cambridge's" not (massachusetts* or boston* or harvard*)) or (canterbury not zealand*) or ("canterbury's" not zealand*) or chelmsford or "chelmsford's" or chester or "chester's" or chichester or "chichester's" or coventry or "coventry's" or derby or "derby's" or (durham not (carolina* or nc)) or ("durham's" not (carolina* or nc)) or ely or "ely's" or exeter or "exeter's" or gloucester or "gloucester's" or hereford or "hereford's" or hull or "hull's" or lancaster or "lancaster's" or leeds* or leicester or "leicester's" or (lincoln not nebraska*) or ("lincoln's" not nebraska*) or (liverpool not (new south wales* or nsw)) or ("liverpool's" not (new south wales* or nsw)) or ((london not (ontario* or ont or toronto*)) or ("london's" not (ontario* or ont or toronto*)) or manchester or "manchester's" or (newcastle not (new south wales* or nsw)) or ("newcastle's" not (new south wales* or nsw)) or norwich or "norwich's" or nottingham or "nottingham's" or oxford or "oxford's" or peterborough or "peterborough's" or plymouth or "plymouth's" or portsmouth or "portsmouth's" or preston or "preston's" or ripon or "ripon's" or salford or "salford's" or salisbury or "salisbury's" or sheffield or "sheffield's" or southampton or "southampton's" or st albans or stoke or "stoke's" or sunderland or "sunderland's" or truro or "truro's" or wakefield or "wakefield's" or wells or westminster or "westminster's" or winchester or "winchester's" or wolverhampton or "wolverhampton's" or (worcester not (massachusetts* or boston* or harvard*)) or ("worcester's" not (massachusetts* or boston* or harvard*)) or (york not ("new york*" or ny or ontario* or ont or toronto*)) or ("york's" not ("new york*" or ny or ontario* or ont or toronto*))))).ti,ab,in. 1909850

63 (bangor or "bangor's" or cardiff or "cardiff's" or newport or "newport's" or st asaph or "st asaph's" or st davids or swansea or "swansea's").ti,ab,in. 77514

64 (aberdeen or "aberdeen's" or dundee or "dundee's" or edinburgh or "edinburgh's" or glasgow or "glasgow's" or inverness or (perth not australia*) or ("perth's" not australia*) or stirling or "stirling's").ti,ab,in. 280945

65 (armagh or "armagh's" or belfast or "belfast's" or lisburn or "lisburn's" or londonderry or "londonderry's" or derry or "derry's" or newry or "newry's").ti,ab,in. 37626

66 or/58-65 3377715

67 (exp africa/ or exp americas/ or exp antarctic regions/ or exp arctic regions/ or exp asia/ or exp australia/ or exp oceania/) not (exp great britain/ or europe/) 3521364

68 66 not 67 [NICE UK Search Filter] 3164655

69 16 and 42 and 57 and 68 656

70 limit 69 to yr="2004 -Current" 517
