# Supplementary material for: Emergency care for young people after self-harm: a realist review protocol
Source: BMJ Open. 2025 Mar 15;15(3):e099554. doi: 10.1136/bmjopen-2025-099554 (PMC11911665; doi:10.1136/bmjopen-2025-099554)
Supplement: Supplementary file 4 [file bmjopen-2025-099554.reviewer_comments.pdf]

PEER REVIEW HISTORY

BMJ Open publishes all reviews undertaken for accepted manuscripts. Reviewers are asked to complete a checklist review form and are provided with free text boxes to elaborate on their assessment. These free text comments are reproduced below.

ARTICLE DETAILS

Title (Provisional)

Emergency care for young people after self-harm: a realist review protocol

Authors

Romeu, Daniel; Ambler, Faye; Brennan, Cathy; Wright, Judy M; Booth, Andrew; Cottrell, David; Guthrie, Elspeth

---

VERSION 1 - REVIEW

**Reviewer**	**1**
**Name**	**Hannigan, Ben**
**Affiliation**	**Cardiff Univ, School of Healthcare Sciences**
**Date**	**02-Feb-2025**
**COI**	**None**

Thank you for this opportunity to read and review this protocol for a realist synthesis in the area of emergency care for young people who have self-harmed. I note the journal's guidance to reviewers accepting invitations to give a view on protocols, which I understand. In this case, I think the paper should be published as it stands. This is a detailed, theory-informed, plan for a comprehensive review in an area of high importance. I look forward to reading more from this project in the future.

**Reviewer**	**2**
**Name**	**Appleton, Rebecca**
**Affiliation**	**University College London**
**Date**	**14-Feb-2025**
**COI**	**None**

This is an extremely comprehensive realist review protocol in an area of research which requires more attention. Whilst I think this is of a high standard and ready for publication, there are a couple of minor additions which would improve clarity of your methodology:

- It would be useful to include details of who the PPI groups are and how they were recruited in the PPI section.

- The search seems relatively broad, encompassing both published and grey international literature, to the point where you may end up with an overwhelmingly high number of sources. I wonder if all exclusion criteria have been considered e.g. explicitly excluding studies of CYP receiving care for self-harm in primary care settings and GP practices? (as these aren't emergency settings)

VERSION 1 - AUTHOR RESPONSE

Reviewer 1:
Thank you for this opportunity to read and review this protocol for a realist synthesis in the area of emergency care for young people who have self-harmed. I note the journal's guidance to reviewers accepting invitations to give a view on protocols, which I understand. In this case, I think the paper should be published as it stands. This is a detailed, theory-informed, plan for a comprehensive review in an area of high importance. I look forward to reading more from this project in the future.

Thank you for such positive feedback, it is greatly appreciated. No changes have been made based on these comments.

Reviewer 2:
This is an extremely comprehensive realist review protocol in an area of research which requires more attention. Whilst I think this is of a high standard and ready for publication, there are a couple of minor additions which would improve clarity of your methodology:

- It would be useful to include details of who the PPI groups are and how they were recruited in the PPI section.

Thank you for the opportunity to provide further detail around PPI. At the start of the PPI section, a paragraph has been added to clarify who the PPI groups are and how they were assembled. An acknowledgements section has also been added to formally thank the groups and the lead representative for the parents and carers' group, who reviewed the protocol in detail and provided invaluable feedback.

- The search seems relatively broad, encompassing both published and grey international literature, to the point where you may end up with an overwhelmingly high number of sources. I wonder if all exclusion criteria have been considered e.g. explicitly excluding studies of CYP receiving care for self-harm in primary care settings and GP practices? (as these aren't emergency settings)

This point is increasingly relevant as we progress through screening of titles and abstracts. The eligibility criteria in Table 1 have been updated to include "studies in non-emergency settings, such as within-hours primary care, inpatient wards and prison settings" as an exclusion criterion. We have also added to the paragraph before the table to acknowledge that the eligibility criteria are likely to be refined and updated as the review progresses, and as programme theories are developed.
